# Tobacco-Derived Nicotine Pouch Brands and Marketing Messages on Internet and Traditional Media: Content Analysis

**DOI:** 10.2196/39146

**Published:** 2023-02-15

**Authors:** Pamela M Ling, Mary Hrywna, Eugene M Talbot, M Jane Lewis

**Affiliations:** 1 Center for Tobacco Control Research and Education and Division of General Internal Medicine University of California San Francisco San Francisco, CA United States; 2 Rutgers Center for Tobacco Studies Rutgers Biomedical and Health Sciences New Brunswick, NJ United States; 3 Department Health Behavior, Society & Policy Rutgers School of Public Health Piscataway, NJ United States

**Keywords:** nicotine pouch, marketing, tobacco industry, web-based advertising, advertising, advertisement, smoking, tobacco, nicotine, smoker, addiction, industry, industrial, economic, economy, commercial, commerce, consumer

## Abstract

**Background:**

Nicotine pouches and lozenges are increasingly available in the United States, and sales are growing. The brands of nicotine pouch products with the largest market share are produced by tobacco companies.

**Objective:**

The aim of this study is to examine the marketing of 5 oral nicotine products sold by tobacco companies.

**Methods:**

Internet, radio, television, print, and web-based display advertisements between January 2019 and March 2020 for 6 brands of nicotine pouches and lozenges were identified through commercially available marketing surveillance systems supplemented by a manual search of trade press and a review of brand websites. A total of 711 advertisements (122 unique) were analyzed to identify characteristics, themes, marketing strategies, and target audiences, and qualitatively compared by brand. All 5 brand websites were also analyzed. Coders examined the entirety of each advertisement or website for products, marketing claims, and features and recorded the presence or absence of 27 marketing claims and lifestyle elements.

**Results:**

All 6 brands of nicotine pouch products spent a total of US $11.2 million on advertising in 2019, with the most (US $10.7 million) spent by the brand Velo, and 86.1% (n=105) of the unique advertisements were web-based. Of the 711 total nicotine pouch advertisements run in 2019, the 2 brands Velo (n=407, 57%) and ZYN (n=303, 42%) dominated. These brands also made the greatest number of advertising claims in general. These claims focused on novelty, modernity, and use in a variety of contexts, including urban contexts, workplaces, transportation, and leisure activities. Of the 122 unique advertisements, ZYN’s most common claims were to be “tobacco-free,” featuring many flavors or varieties, and modern. Velo was the only brand to include urban contexts (n=14, 38.9% of advertisements) or freedom (n=8, 22.2%); Velo advertisements portrayed use in the workplace (n=15, 41.7%), bars or clubs (n=5, 13.9%), leisure activities (n=4, 11.1%), transportation (n=4, 11.1%), sports (n=3, 8.3%), cooking (n=2, 5.6%), and with alcohol (n=1, 2.8%). Velo and ZYN also included most of the images of people, including women and people of color. The 36 Velo ads included people in advertising in 77.8% (n=28) of advertisements, and of those advertisements with identifiable people, 40% (n=4) were young adults and 50% (n=5) were middle-aged. About one-third (n=11, 35.5%) of the 31 unique ZYN advertisements included people, and most identifiable models appeared to be young adults. Brands such as Rogue, Revel, Dryft, and on! focused mainly on product features. All nicotine pouch products made either tobacco-free, smoke-free, spit-free, or vape-free claims. The most common claim overall was “tobacco-free,” found in advertisements from Rogue (1/1, 100%), ZYN (30/31, 96.8%), Velo (19/36, 52.8%), and Dryft (1/3, 33.3%), but not Revel.

**Conclusions:**

Nicotine pouches and lozenges may expand the nicotine market as tobacco-free claims alleviate concerns about health harms and advertising features a greater diversity of people and contexts than typical smokeless tobacco advertising. The market leaders and highest-spending brands, ZYN and Velo, included more lifestyle claims. Surveillance of nicotine pouch marketing and uptake, including influence on tobacco use behaviors, is necessary.

## Introduction

In 2009, the tobacco company Reynolds American Inc acquired the company Niconovum AB, which produced nicotine gum, and in 2012, the company began test-marketing Zonnic brand nicotine pouches in convenience stores and gas stations [1]. New tobacco-free nicotine pouch products marketed primarily as alternatives to other tobacco products emerged in the United States several years later. Sales of nicotine pouches in the United States increased substantially between 2016 and 2020 [2], and almost 30% of adult smokers were aware of nicotine pouches in 2021 [3]. A 2022 study of social media posts on Reddit found the number of posts related to oral nicotine pouches increased between 2019 and 2021, and the most common topics were sharing product information and user experiences [4]. Nicotine pouches typically contain nicotine, binders, and flavors in a porous pouch that is placed on the oral mucosa [5] to allow nicotine absorption similar to smokeless tobacco products [6]. As of 2021, the most popular nicotine pouch product brands available in the United States were owned or distributed by companies that also manufacture and sell cigarettes, cigars, or smokeless tobacco products. Swedish Match introduced its pouch product, ZYN, in test markets in 2014. This was followed by the introduction of Dryft (Kretek) and on! (Philip Morris) in 2016. In 2018, Rogue nicotine pouches, gums, and lozenges were introduced by NicoGen pharma with national rollouts in 2019; Swisher International began to distribute Rogue oral nicotine products late in 2019. Reynolds American Incorporated (RAI) introduced a nicotine lozenge (Revel) early in 2019, followed by a nicotine pouch, Velo, later that year. Subsequently, in 2020, RAI rebranded Revel lozenges under the Velo brand name; it also acquired Dryft pouches and rebranded them under Velo (Table 1).

**Table 1:** Nicotine pouch brands available in the United States in 2019-2020.

Brand	Manufacturer	Nicotine levels	Flavors	Launched
ZYN 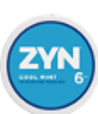	Swedish Match	3 mg 6 mg	Cool mint, peppermint, wintergreen, spearmint, cinnamon, coffee, citrus; unflavored includes smooth and chill	2014 in test markets; 2019 nationally
on! 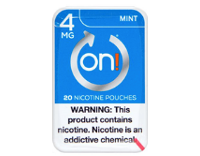	Altria (formerly Burger Sohne)	1.5 mg 2 mg 3.5 mg 4 mg 8 mg	Berry, cinnamon, citrus, coffee, wintergreen; unflavored includes original	2016
Rogue 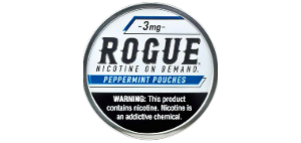	Swisher International (formerly Nicogen Pharma)	3 mg 6 mg	Apple, cinnamon honey lemon, mango, peppermint, wintergreen	2018
Velo and Velo Max (formerly Dryft) 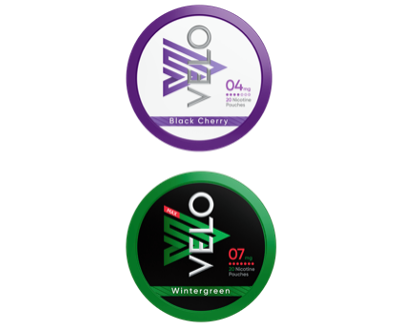	RAI^a^ -RJ Reynolds Vapor Company (Dryft brand formerly owned by Kretek International)	2 mg 4 mg 7 mg	Black cherry, citrus burst, cinnamon, coffee, dragon fruit, peppermint, spearmint, wintergreen	2016 (Dryft); 2019 (Velo); 2020 (Dryft rebranded as Velo Max)

^a^RAI: Reynolds American Incorporated.

Swedish Match (which produces the ZYN brand) had the largest market share, peaking at 92.6% in July 2019 and 78.7% in June 2020 by units sold, which were much greater than the other nicotine pouch products [2]. Nicotine pouches are available in a wider variety of flavors (eg, mango, black cherry, citrus, and dragon fruit) compared to Food and Drug Administration (FDA)–approved nicotine replacement gum or lozenges. There is a limited body of literature on nicotine pouch products, and most papers focus on the toxicant or nicotine content of the products [6-9]. While nicotine pouch products may have lower levels of toxicants than combustible cigarettes or smokeless tobacco products, they may also perpetuate or prolong nicotine addiction or act as a means for young people to initiate nicotine use [10]. In studies of nicotine pouch brands sold in the United States, the maximum nicotine content was found to be under 12 mg/pouch [8,10], but in a convenience sample consisting of 46 different pouch samples purchased in web-based shops, researchers in Germany found that nicotine contents ranged from 1.79 to 47.5 mg/pouch [11]. One paper based on consumer data reported that nontobacco users had low interest in the ZYN pouches, and most users of ZYN were former tobacco users; notably, this paper was supported by the tobacco company Swedish Match, which has a financial interest in publicizing the product positioning as being for adult tobacco users [12].

In addition to the toxicant and nicotine content characteristics of the products, the public health impact of nicotine pouch products depends on the marketing and advertising, which influence their uptake and patterns of use [9]. There have been few studies on the marketing of nicotine pouch products. There has been a documented shift in advertising expenditures within the smokeless tobacco product category between 2018 and 2020, with the majority of recent promotional spending on nicotine pouches as compared to conventional smokeless tobacco and snus [13]. One paper reviewed 50 pieces of direct mail advertising for 3 brands of nicotine pouch products (Velo, on!, and Revel) and described the basic claims in these advertisements: 90% claimed to be an alternative to another tobacco product like cigarettes or smokeless tobacco, 70% claimed that the product could be used anywhere, and almost half contained claims that oral nicotine was spit-free (58%), smoke-free (42%), or free of tobacco leaf (42%) [14]. However, this analysis was limited to direct mail for 3 brands and did not include the market leader, ZYN. More recently, Duan and colleagues [15] used advertising data from 2019 through 2021 to examine how nicotine pouch brands including Velo, on!, and ZYN were marketed and found themes such as freedom, brand, and flavor were most prominent. For this study, we conducted a content analysis that addresses 6 brands of nicotine pouch products produced by tobacco companies and includes web-based, website, radio, and television advertising channels used in 2019. In addition, we reviewed the trade press to identify business-to-business advertisements from 2019. The overall goal of the study was to describe messages to sell nicotine pouch products in 2019 while comparing the different brands’ positioning in messages directed at consumers and tobacco businesses.

## Methods

### Overview

We extracted data collected by Kantar Media from the calendar year (January to December) 2019, including expenditures on web-based advertisements, radio, television, and print for 6 nicotine pouch brands (ZYN, Velo, Dryft, Rogue, Revel, and on!). For web-based display advertisements, Kantar provided monthly reports from January to December 2019, and each monthly report included a ZIP file containing copies of all the advertisements. Each copy was reviewed to identify unique advertisements, defined as featuring a distinct combination of image and text; advertisements that varied only by the size of image, text, or layout (eg, moving the slogan from the bottom to the side of the image) were considered not unique. In addition to web-based display advertisements, Kantar provided monthly reports of all radio and television ad runs from January to December 2019 and provided a ZIP file with copies of the advertisements. From these files, 4 unique radio advertisements and 4 unique television advertisements were extracted and downloaded as MP4 files.

In addition to the data acquired from Kantar, we searched the database What Runs Where [16] to identify additional web-based advertisements not included in the Kantar data. We identified advertising in the United States between May 2019 and February 2020 for the 6 oral nicotine product brands of interest and downloaded the list of advertisements, images, and metadata. We identified unique web-based advertisements using the same criteria as the Kantar data set. We also accessed the brand websites for each of the 5 nicotine pouch brands that had brand websites. In addition, we accessed the monthly archives from February 2019 to March 2020 for 2 web-based trade magazines, Convenience Store News & Petroleum, and Convenience Store Decisions, and reviewed each issue for nicotine pouch advertising. Pages that contained advertisements for pouch nicotine products were copied and saved as electronic files. The total combined data set consisted of 711 advertisements: 287 local radio, 211 web-based display advertisements, 99 mobile web advertisements, 42 spot television, 35 web-based video, 13 cable television, 9 national spot radio, 7 business-to-business advertisements, 6 outdoor, and 2 syndicated. Within the data set, we identified 122 unique advertisements: 105 web-based displays, 4 television, 4 radio, 5 brand websites, and 9 advertisements from the trade press.

### Coding Guide and Development Procedures

A coding guide developed for electronic cigarette websites [17] was adapted for nicotine pouch websites, web-based displays, and other advertising. The guide was tested iteratively on each type of advertisement, and investigators discussed the definitions, discrepancies, or missing concepts, followed by guide revision. When consensus was reached, 3 investigators double-coded all advertisements. Coders examined the entirety of each advertisement or website for products, marketing claims, and features. They recorded the availability of product features and the presence or absence of 27 marketing claims and lifestyle elements. When people were present in the advertisement and a face was visible, the demographic characteristics of the person were coded. Discrepancies in coding were reviewed iteratively by the team and discussed, and the guide was repeatedly revised to include new categories and generate consistent definitions until reliability was established. On the test sites, reliability was ĸ=0.87.

### Ethical Considerations

This study is exempt from human subjects research review as no human subjects were involved.

## Results

### Advertising Spending

Spending on advertising for nicotine pouches in 2019 by the brand was reported by Kantar Media for the internet, radio, television, and other channels. A total of US $11.2 million was spent, with US $5.5 million (49.1%) spent on cable TV advertisements, followed by US $1.8 million (16.1%) on local radio and US $1.2 million (10.7%) on national radio, and US $748,000 on internet display advertisements. The majority of spending took place in the fall of 2019 after RAI launched Velo. Velo also dominated in terms of dollars spent: Velo spent US $10.7 million of the US $11.2 million estimated total spending on advertising. By ad count, most advertising was for 2 brands: Velo, which ran 407 (57.2%) of the advertisements, and ZYN, which ran 303 (42.6%) of the advertisements in the data set; most advertisements ran on local radio (n=287, 40.4%), internet display (n=211, 29.7%), and mobile web (n=99, 13.9%).

### Marketing Claims in Nicotine Pouch Advertising

Table 2 summarizes the frequency with which claims appeared in advertising for each of the 5 nicotine pouch brands that were advertised on TV, radio, print, and the internet. Each of the brands included some reference to other tobacco products in their advertising, although the specific product varied by brand. The most common claim was “tobacco-free,” which was included in advertisements from Rogue (1/1, 100%), ZYN (30/31, 96.8%), Velo (19/36, 52.8%), and Dryft (1/3, 33.3%), but not Revel. With regard to specific tobacco products, 31.4% (16/51) of Revel and 16.7% (6/36) of Velo advertisements referred to cigarettes, 13.9% (5/36) of Velo and 5.9% (3/51) of Revel advertisements referred to e-cigarettes, and 8.3% (3/36) of Velo and 6.5% (2/31) of ZYN advertisements referred to smokeless tobacco. Most nicotine pouch brands included “smoke-free” and “spit-free” claims in at least one ad, but only Dryft included the claim “vape-free.” Only ZYN included a reference to nicotine salts, which appeared in 9.7% (3/31) of its advertisements.

**Figure 1:**
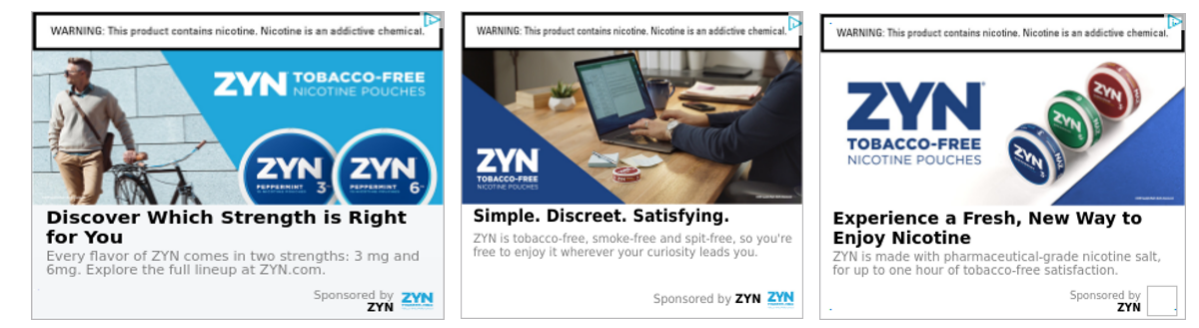
Examples of ZYN web-based display advertising featuring transportation, workplace, and product variety images.

The different pouch brands emphasized several marketing and lifestyle claims in their advertising. ZYN advertisements’ most common claims emphasized product features (eg, product choice including a variety of flavors, modern or high-tech features, and use of pharmaceutical terms) and use in a variety of situations (eg, ability to use anywhere, use on transportation, in the workplace, in bars or clubs, with alcohol, and during social interactions). About one-third (11/31, 35.5%) of ZYN advertisements included people, and 81.8% (n=9) of those advertisements included a person’s face. Of advertisements with identifiable people, 55.6% (n=5) of models appeared to be young adults and 22.2% (n=2) appeared to be middle-aged; 100% (n=9) included male models and 44.4% (n=4) included female models; 77.8% (n=7) of models appeared to be White people, and 22.2% (n=2) were of indeterminate race. Examples of ZYN advertising reflecting these claims are included in Figure 1.

**Figure 2:**
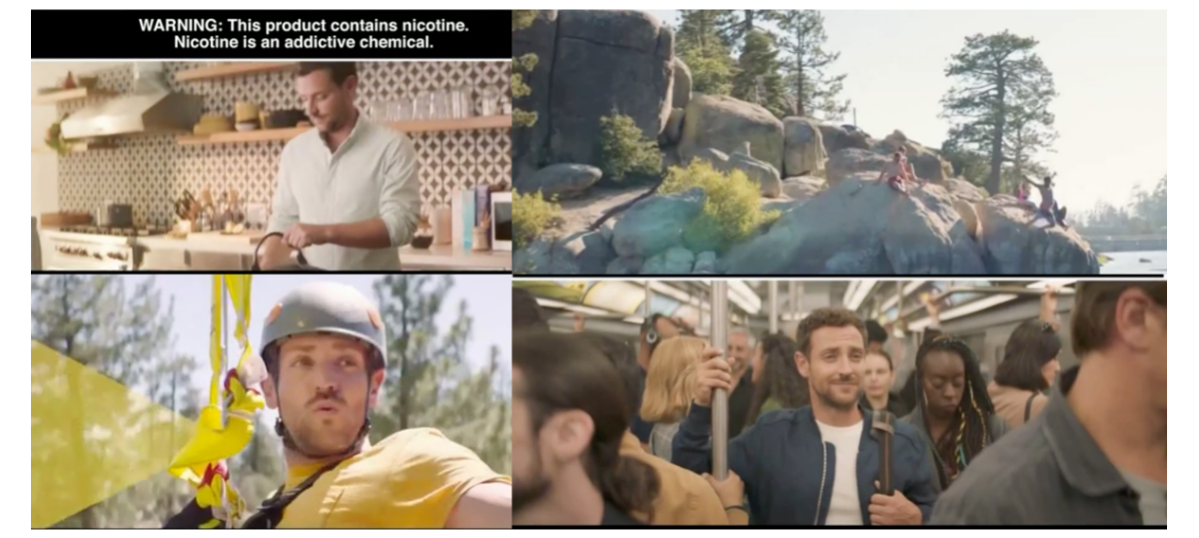
Examples (screen shots) from a web-based Velo advertising video featuring recreational activities (swimming, zip-lining), urban contexts, and transportation (riding on the subway).

Similar to ZYN, Velo advertisements emphasized the modern aspects of the product, its convenience, and its ability to be used anywhere and in social situations. Velo was the only brand to include urban contexts in its 36 advertisements (n=14, 38.9%) or freedom (n=8, 22.2%). In addition, 41.7% (n=15) of Velo advertisements portrayed use in the workplace, while bars and clubs (n=5, 13.9%), leisure activities (n=4, 11.1%), transportation (n=4, 11.1%), sports (n=3, 8.3%), cooking (n=2, 5.6%), and alcohol (n=1, 2.8%) were also included. Of all brands, Velo included people in its advertising the most frequently (n=28, 77.8% of advertisements), though faces were visible in only 25% (n=7) of those advertisements. In advertisements with identifiable people, 40% (n=4) were young adults and 50% (n=5) were middle-aged, 70% (n=7) included male models, 30% (n=3) included female models, 55.6% (n=5) appeared to be White people, 11.1% (n=1) appeared to be Black people, 11.1% (n=1) appeared to be Hispanic or Latinx people, and 44.4% (n=4) appeared to be of indeterminate race or ethnicity. Velo spent the most advertising dollars on television, and the television advertisements included the most varied lifestyle context imagery, including quick cuts between urban, office, transportation, and recreational activities (Figure 2).

**Figure 3:**
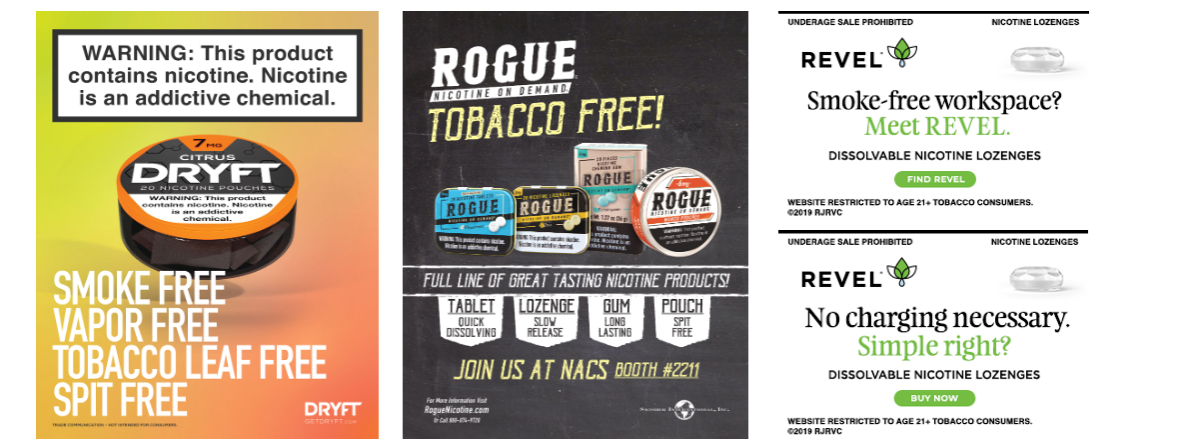
Business-to-business advertisements for Dryft and Rogue and web-based display advertisements for Revel featuring references to smoking, tobacco products, and e-cigarettes.

In contrast to ZYN and Velo advertising, the advertising for the Dryft, Rogue, and Revel brands focused mainly on product features. None of these advertisements featured people in imagery. Both Dryft and Rogue brands included the theme of “choice,” which was defined in the code book as “statements that suggest a user has many options or choices or varieties of products, flavors, taste or strengths, statements like ‘you have a choice’, or presenting multiple product options…presenting a lot of choices.” Dryft advertising included smoke-free, spit-free, vape-free, and tobacco-free wording, along with the claim of no secondhand smoke. Rogue advertisements all included claims of convenience and choice, and Rogue was the only brand that used food safety terms such as “food grade ingredients,” which were present in 100% (1/1) of Rogue advertisements. The 51 Revel ads emphasized use anywhere, convenience, and smoke-free, and the text referred to a variety of lifestyle situations for use, such as urban contexts (n=4, 7.8%), workplace (n=10, 19.6%), transportation (n=4, 7.8%), and references to smoking or situations where one cannot smoke (n=8, 15.7%). Examples of these claims are in Figure 3.

**Table 2:** Percent of 2019 advertisements from internet, radio, television, print, and outdoor advertising containing marketing claims and lifestyle elements by nicotine pouch brand.

			ZYN (n=31), n (%)		Velo (n=36), n (%)		Dryft (n=3), n (%)		Rogue (n=1), n (%)		Revel (n=51), n (%)		Overall (n=122), n (%)
**Marketing claims**													
	Refers to cigarettes	1 (3.2)		6 (16.7)		0 (0.0)		0 (0.0)		16 (31.4)		23 (18.9)	
	Refers to e-cigarettes	0 (0.0)		5 (13.9)		0 (0.0)		0 (0.0)		3 (5.9)		8 (6.6)	
	Refers to ST^a^	2 (6.5)		3 (8.3)		0 (0.0)		0 (0.0)		0 (0.0)		5 (4.1)	
	Smoke-free	3 (9.7)		5 (13.9)		1 (33.3)		0 (0.0)		4 (7.8)		13 (10.7)	
	Spit-free	3 (9.7)		1 (2.8)		1 (33.3)		1 (100.0)		0 (0.0)		6 (4.9)	
	Vape-free	0 (0.0)		0 (0.0)		1 (33.3)		0 (0.0)		0 (0.0)		1 (0.8)	
	Tobacco-free	30 (96.8)		19 (52.8)		1 (33.3)		1 (100)		0 (0.0)		51 (41.8)	
	Nicotine salts reference	3 (9.7)		0 (0.0)		0 (0.0)		0 (0.0)		0 (0.0)		3 (2.5)	
	Includes warnings	27 (87.1)		34 (94.4)		2 (66.7)		0 (0.0)		51 (100)		114 (93.4)	
	Discounts offered	0 (0.0)		4 (11.1)		0 (0.0)		0 (0.0)		3 (5.9)		7 (5.7)	
	Smoke-free policy reference	0 (0.0)		1 (2.8)		0 (0.0)		0 (0.0)		4 (7.8)		5 (4.1)	
	Use anywhere	5 (16.1)		20 (55.6)		0 (0.0)		0 (0.0)		17 (33.3)		42 (34.4)	
	Freedom	0 (0.0)		8 (22.2)		0 (0.0)		0 (0.0)		0 (0.0)		8 (6.6)	
	Modern or high-tech	6 (19.4)		11 (30.6)		0 (0.0)		0 (0.0)		0 (0.0)		17 (13.9)	
	Convenience	2 (6.5)		4 (11.1)		0 (0.0)		1 (100.0)		11 (21.6)		18 (14.8)	
	Choice	8 (25.8)		2 (5.6)		1 (33.3)		1 (100.0)		0 (0.0)		12 (9.8)	
	Urban context	0 (0.0)		14 (38.9)		0 (0.0)		0 (0.0)		4 (7.8)		18 (14.8)	
	No SHS^b^	0 (0.0)		0 (0.0)		1 (33.3)		0 (0.0)		0 (0.0)		1 (0.8)	
	Food safety terms	0 (0.0)		0 (0.0)		0 (0.0)		1 (100.0)		0 (0.0)		1 (0.8)	
	Pharmaceutical terms	3 (9.7)		0 (0.0)		0 (0.0)		0 (0.0)		0 (0.0)		3 (2.5)	
	Social interactions	2 (6.5)		5 (13.9)		0 (0.0)		0 (0.0)		0 (0.0)		7 (5.7)	
	Link to website	14 (45.2)		35 (97.2)		3 (100.0)		1 (100.0)		18 (35.3)		71 (58.2)	
**Lifestyle elements**													
	Leisure	0 (0.0)		4 (11.1)		0 (0.0)		0 (0.0)		0 (0.0)		4 (3.3)	
	Bars or clubs	2 (6.5)		5 (13.9)		0 (0.0)		0 (0.0)		0 (0.0)		7 (5.7)	
	Workplace	3 (9.7)		15 (41.7)		0 (0.0)		0 (0.0)		10 (19.6)		28 (23)	
	Sports	0 (0.0)		3 (8.3)		0 (0.0)		0 (0.0)		0 (0.0)		3 (2.5)	
	Transportation	6 (19.4)		4 (11.1)		0 (0.0)		0 (0.0)		4 (7.8)		14 (11.5)	
	Cooking	0 (0.0)		2 (5.6)		0 (0.0)		0 (0.0)		0 (0.0)		2 (1.6)	
	Smoking	0 (0.0)		0 (0.0)		0 (0.0)		0 (0.0)		8 (15.7)		8 (6.6)	
	Alcohol use	2 (6.5)		1 (2.8)		0 (0.0)		0 (0.0)		0 (0.0)		3 (2.5)	

^a^ST: smokeless tobacco.

^b^SHS: secondhand smoke.

### Other Features

All brands except Rogue included warning labels on the majority of advertisements, which appeared on 66.7% (2/3) of Dryft, 87.1% (27/31) of ZYN, 94.4% (34/36) of Velo, and 100% (51/51) of Revel advertisements. Offers for discounts were present but relatively rare in nicotine pouch advertising, appearing only in the Velo (4/36, 11.1%) and Revel (3/51, 5.9%) advertisements. A minority of advertisements referred to smoke-free policies, mainly Revel (4/51, 7.8%) and Velo (1/36, 2.8%). All of the brands included links to their websites, particularly Dryft (3/3, 100%), Rogue (1/1, 100%), and Velo (35/36, 97.2%), and less frequently, ZYN (14/36, 45.2%) and Revel (18/51, 35.3%).

### Brand Website Marketing Claims

Since each brand had only 1 website (N=5), the website content is presented separately. All 5 pouch nicotine products had websites for analysis. The on! brand of nicotine pouches only had website advertising (no other channels such as radio or print) in the data set, and Revel did not have a website. All 5 websites included age-gating, such as a pop-up window where visitors were asked if they were 21 years or older, but only ZYN required registration to access the site; this registration did not include ID verification. All sites included warning labels. All 5 brands include the wording smoke-free, spit-free, and tobacco-free with references to cigarettes; all sites except ZYN included references to e-cigarettes and smokeless tobacco. All sites offered a variety of flavors and nicotine strengths (Table 1). Dryft featured the largest number of flavors and the highest nicotine content. Similar to the other advertising channels, the ZYN and Velo websites had the greatest number and variety of marketing claims (all claims in Table 2 were present on the Velo website, and most were present on the ZYN website), and all lifestyle elements were present on both the ZYN and Velo websites. Rogue had fewer claims but included freedom, the ability to use it anywhere, control, social status, and easing social interactions, as well as food safety terms and the assurance that the product was made in the United States. The 2 lifestyle elements on the Rogue website were workplace environments and enhancing pleasurable activities. In contrast, on! and Dryft made relatively few marketing claims on their websites; claims were limited to use anywhere and in urban contexts. The workplace was the only lifestyle element present on the on! website and Dryft only included leisure and pleasurable activities. ZYN, Velo, and Dryft were the only brand websites that included images of people; ZYN and Velo included images of groups of people, while Dryft’s images were of single people. Only Velo offered discounts on the website, and only Velo referred to community empowerment.

## Discussion

### Principal Findings

This content analysis of nicotine pouch product advertising adds to the literature by addressing a variety of product brands and advertising channels, with a deliberate comparison of messages by brand. We found distinct differences among the different pouch product brands, with more lifestyle-focused messaging in the 2 most heavily advertised brands, ZYN and Velo. The other brands’ advertising focused more on product characteristics. ZYN and Velo are also the products that have been on the market the longest and the gradual shift in advertising emphasis from the product characteristics to the user and lifestyle is consistent with prior studies of cigarette advertising describing the introduction of new products [18]. Similar to previous work [15], we found Velo accounted for more dollars spent and more ad occurrences compared to other brands. In addition, Velo and ZYN advertisements included more imagery relative to other brands’ advertisements.

It is worth noting that since this content analysis was conducted, the number and variety of pouches and other oral nicotine products have continued to increase, often within a single brand. Two brands we studied, Revel and Dryft, were subsequently acquired by RAI and rebranded as Velo products, greatly extending this brand’s number of nicotine products, flavors, and nicotine strengths. The Rogue brand also offers several oral nicotine products—pouches, lozenges, and gum—under a single brand. In 2021, the nicotine gum brand Lucy offered nicotine gum, lozenges, and pouches under the same brand [19]. Some of the nicotine pouch products include claims to be manufactured from synthetic or “tobacco-free nicotine,” mirroring claims found in e-cigarettes and liquids [20,21]. The use of terms such as “pharmaceutical grade nicotine” and ingredients labeled “generally recognized as safe by FDA” in these products may also alleviate concerns about the health harms of tobacco product use.

While some of these products resemble nicotine replacement therapy, all of them are notably marketed without overt claims to be smoking cessation aids. As tobacco companies promote their nicotine pouch products with their cigarette brands [22], this might encourage smokers to switch to oral nicotine products to support smoking cessation, although if the products are used primarily to maintain nicotine use in smoke-free environments, they might prolong rather than reduce combustible cigarette use. In addition, companies selling most of the major tobacco-derived nicotine pouch brands, including ZYN, on!, Velo, and Rogue, have submitted Premarket Tobacco Product Applications to the FDA. The current evidence base lacks data on the use of nicotine pouch products for smoking cessation. It will be important to monitor nicotine pouch brands, marketing, and use over time, as well as its influence on other tobacco use patterns. Nicotine pouch products may benefit cigarette smokers and smokeless tobacco users who decide to exclusively switch to a tobacco-free product. However, exclusive switching is not encouraged by current marketing messages, which encourage the use of nicotine pouches in many environments where smoking is not allowed. These marketing messages may encourage the use of both cigarettes and nicotine pouches.

The uptake of nicotine pouches among naïve tobacco users is also an unintended consequence of an innovative, appealing nicotine product with limited regulation. The variety of demographic characteristics in the people portrayed in ZYN and Velo brand advertising and the inclusion of urban contexts, offices, and lifestyle activities differ from typical smokeless tobacco marketing [23]. This raises the question of whether the marketing messages for these products are intended to expand the nicotine market. In addition, Velo pouches have been found to have a lower nicotine content [10], which may make them easier for novices to adopt. One important marketing channel that may affect youth uptake is social media, which was not included in this analysis. A content analysis of over 600 Reddit posts about nicotine pouch products found that over half of the posts expressed positive sentiments about oral nicotine pouches [4]. Formal content analysis of social media marketing for oral nicotine products with attention to youth appeal is warranted. The diversification of audiences from White men to include women and people of color, which was first explored with little success with traditional smokeless tobacco products [24], may be more successful with nicotine pouch products.

Oral nicotine is a rapidly evolving product category, and we were limited in our ability to capture nicotine pouch brands still emerging on the market, so our work provides only a snapshot of the market during the time period of interest. However, this study extends previous work on oral nicotine marketing by including ZYN, the overwhelming market leader in the category since 2016. In addition, we coded for a wide variety of product characteristics and marketing claims across several media platforms. It will be important to monitor changes in nicotine pouch marketing over time as new products emerge, synthetic nicotine products expand, and local, state, and federal policies are enacted. In addition, studies are needed to address the impact of exposure to these marketing messages on consumer perceptions and behavior, as well as the impact of nicotine pouch product use on other tobacco product use in the population.

### Conclusions

This formal content analysis of nicotine pouch brand marketing highlights substantial investments in advertising, including users and contexts different from typical smokeless tobacco marketing, particularly among the brands with the largest market share. These claims, along with those that evoke perceptions of increased safety and differentiate nicotine pouches from other tobacco products, have the potential to expand the nicotine market. Future research should address messaging that may have more claims that appeal to youth, including social media channels and nicotine pouch brands that are smaller or not associated with major tobacco companies. Continued surveillance of new products, marketing claims, population uptake, and impact on tobacco and nicotine use behaviors is warranted.

## Abbreviations

FDA: Food and Drug Administration
RAI: Reynolds American Incorporated

## Data Availability

Kantar data are proprietary and are not allowed to be made public. Data for this study may be requested and purchased from Kantar.
